# Supplementary material for: Acute strength exercise training impacts differently the HERV-W expression and inflammatory biomarkers in resistance exercise training individuals
Source: PLoS One. 2024 May 16;19(5):e0303798. doi: 10.1371/journal.pone.0303798 (PMC11098355; doi:10.1371/journal.pone.0303798)
Supplement: Attachment — Submitted filename: rebuttal letter.doc [file pone.0303798.s002.doc]

Luiz Henrique da Silva Nali, PhD

Pós graduação em Ciências da Saúde

Universidade de Santo Amaro

Av Dr Eneas de Siqueira Neto, 340 – São Paulo

lnali@prof.unisa.br

March, 12^th^ 2024

Professor Mathew Matthew Cserhati, Ph.D

Academic Editor, Plos ONE

Dear Professor,

We are pleased for the evaluation of our paper by the reviewer and the editor. In this letter we bring answers to all the questions raised by them. We are also submitting the reviewed version of the manuscript for your consideration.

Editor comments:

1. Please ensure that your manuscript meets PLOS ONE's style requirements, including those for file naming. The PLOS ONE style templates can be found at

<https://journals.plos.org/plosone/s/file?id=wjVg/PLOSOne_formatting_sample_main_body.pdf> and

<https://journals.plos.org/plosone/s/file?id=ba62/PLOSOne_formatting_sample_title_authors_affiliations.pdf>

*Authors’ Answer: We have now uploaded the updated versions of manuscript files according to the provided templates*

2. Please note that funding information should not appear in any section or other areas of your manuscript. We will only publish funding information present in the Funding Statement section of the online submission form. Please remove any funding-related text from the manuscript.

*Authors’ Answer: We have now removed this information of the manuscript as requested.*

3. Thank you for stating the following financial disclosure:

"Fundação de Amparo à Pesquisa do Estado de São Paulo (FAPESP), grants # 2023/08773-0 and 2020/11619-5"

Please state what role the funders took in the study. If the funders had no role, please state: "The funders had no role in study design, data collection and analysis, decision to publish, or preparation of the manuscript."

If this statement is not correct you must amend it as needed.

Please include this amended Role of Funder statement in your cover letter; we will change the online submission form on your behalf.

*Authors’ Answer: The funders had no role in study design, data collection and analysis, decision to publish, or preparation of the manuscript. We have also included this statement in the updated cover letter*

4. We note that your Data Availability Statement is currently as follows: All relevant data are within the manuscript and its Supporting Information files

Please confirm at this time whether or not your submission contains all raw data required to replicate the results of your study. Authors must share the “minimal data set” for their submission. PLOS defines the minimal data set to consist of the data required to replicate all study findings reported in the article, as well as related metadata and methods

*Authors’ Answer: We are submitting at this time all the raw data required to replicate the results of the study.*

Additional Editor Comments:

Acute strength exercise training impacts differently the HERV-W expression and inflammatory biomarkers in resistance exercise training individuals

P ONE-D-24-05279

Smaller comments:

1. Line 54: “Inflammation is a vital and evolutionary conserved process…” Evolution means change, and conservation means stasis, so this term–though widespread–is an oxymoron. Use a better term, something like “ubiquitous”.

*Authors’ Answer: We thank the editor for the term suggestion, it was changed as suggested.*

2. 58: well-coordinate: do you mean well-coordinated?

*Authors’ Answer: Yes, we do. The sentence was corrected accordingly. Thank you.*

3. 65: do you mean (IL-6) ?

*Authors’ Answer: Yes, it was a typo and we apologize for that.*

4. 73: that rises promptly …

5. 83: the transcription of

6. 87: Mendelian…

*Authors’ Answer: We thank for the editor revision, and we apologize for the grammar mistakes, we have thoroughly reviewed the text for any more typos and grammar errors.*

7. 304: what does p=0.0XX mean? Did you want to write a number in place of the X?

*Authors Answer: We did. In fact, this information was missing in the text, we have added it.*

1. Someone should read the paper and correct the English, there are slight mistakes here and there.

*Authors’ Answer: We thank the editor for point this out. We have thoroughly reviewed the text for any remaining typos and grammar errors. Additionally, we have sent this version to a native English speaker for comprehensive review.*

2. 82-89: The idea that HERV’s are ancient viruses that infected the human genome millions of years ago is contested. There is no direct evidence for this. For example, some researchers have studied the Arc gene, which modulates synaptic plasticity in the brain. The Arc mRNA is able to assemble itself into virus-like capsids that encase RNA, exhibiting traits of retroviral Gag proteins. See Pastuzyn et al., 2018., Cell. These HERV’s instead might be design structures in the genome. This being said, is it not possible that HERV-W and -K are actually integral parts of the human genome that are activated during physical exercise?

*Authors’ Answer: We thank the editor for this consideration and for the opportunity to clarify this interesting hypothesis.*

*We would like to answer the second question first: “*…is it not possible that HERV-W and -K are actually integral parts of the human genome that are activated during physical exercise?” *Indeed, HERV-K and HERV-W are integral parts of the human genome, along with other endogenous retroviral families, collectively constituting approximately 8% of the human genome. Except for a few polymorphic proviruses from K family, all human being has the same HERV composition, which allow us to say that ERVs make us humans. So the Editor is right, the HERV-W is working as a human (though domesticated) gene that is reactivated under certain conditions.*

*The origin of these retroviral sequences is object of study of many researchers. While the idea of ancestral retroviral endogenization events occurring millions of years ago seems intriguingly, previous studies, using comparative genomics and evolutionary approaches were able to estimate time of these insertions into our ancestral’s genome. In the same line of investigation, several papers elegantly described the dynamics of the distribution of these ERVs among different species*[1–6]*. Interestingly, it was also observed that during host speciation events, the gain/loss and fixation of the proviruses were dramatically impacted by demographic conditions* [7]*. Using orthologs elements, a team from Imperial College London demonstrated that a particular member of ERV-L, one of the most ancient integrated families, predates the divergence of the placental mammals, and conserved fragments are present also in primates*[8]*. But not all ERVs are that old. Some families are specific from primates, as HERV-W and the newest one, HERV-K, present only in Old World primates since they integrated in the ancestral genome after the divergence of the New and Old World primates. Still regarding HERV-K, some of them, such as HERV-K-113 and HERV-K 115 frequency vary depending on the population, being present in up to 23% and 35% of the population*[9]*.*

*Maybe the best example, in real time, of the endogenization process are the koala retroviruses. Koala Retrovirus (KoRV) is an important exogenous retrovirus that represent a threat to Koalas. This virus can be either found in genome of some Koala’s population as an endogenized element, verified by high proviral load, or in the exogenous and infectious condition. These conditions are found in particular areas in the island of northeastern coast of Australia*[10]*. Phylogenetic analysis of KoRV-A LTRs revealed that this integration event had begun around 50.000 years ago and is still in course* [11]*. This piece of evidence is clarifying how this complex event is occurring in a specific condition.*

*Finally, to our understanding, Patuzyn et al., 2018 does not contest HERVs as ancient retroviral integrations but rather describes that Arc possesses molecular properties resembling those of retroviral Gag proteins. In their work, they demonstrated that Arc is derived from a vertebrate lineage of Ty3/gypsy, which is a retrotransposon (considered ancestors of modern retroviruses), not a retrovirus. And, as is the case with all vertebrate genomes, there are several mobile elements from different classes (ranging from DNA transposons to RNA long and short elements such as LINEs and SINEs) as well as retrotransposons and retroviruses. HERVs are integral part of the genome and as described in lines 74-79 (updated version), events of gene domestication* [12] *occurred throughout the evolution and are crucial gene regulation*[13,14] *and also for the human physiology.*

3. 306-7: there was a positive correlation between IL-10 (T0) and TNFa (T1), but the correlation is moderate at best. Also, why did you compare two different genes at two different time points? Why not IL-10 (T0) versus TNFa (T0) and IL-10 (T1) versus TNFa (T1)?

*Authors Answer: We thank the editor for this consideration. Actually, we have performed the correlation analysis in all possible combinations, including the ones highlighted by the editor. However we did not observe any significant positive or negative correlation between these cytokines’ concentration in the specific time points. We have added this information in the results section (lines 278-281)*

4. 338: Why is it significant that HERV-W is induced, but not HERV-K?

*Authors’ Answer: Our findings are interesting since supports that HERV-W play a role in the muscle repair physiology and therefore might be upregulated in this condition, while the newest and most active family- HERV-K (HML-1-10, also analyzed here), is not.* *This is also critical since the expression of one family but not others is not occurring as a consequence of epigenetic changes after cellular stress, caused by local inflammation, but rather, the HERV regulation may be intricately linked to a very specific role in muscular repair. And this make sense since syncytin-1 is responsible for fusogenic events commonly observed in different cell types formation, such as syncytiotrophoblast*[12] *and osteoclasts*[15,16]*. Therefore, since HERV-W is upregulated in a particular exercised individuals, it might be significant to identify this difference. We have highlighted this in the discussion session (lines 354-361)*

5. 480-88: The present study presents interesting information, but adding protein analysis would strengthen your results. Also, if possible, it would be even better if you could follow up with the study participants. Hence, I would like to return your paper for major revisions.

*Authors’ Answer: We agree with the editor, in fact the protein analysis would strengthen our findings and it was discussed as a limitation of the study (lines 485-486) However, there is a lack of data regarding the modulation of HERVs expression in acute physical exercise. We understand that this modulation might change in the next days or weeks, and different scenario might appear, this should certainly be the focus for next studies, we have also brought this matter in the discussion section (lines 491-497)*

**# Reviewer 1**

*We have omitted the reviewer`s response to Plos ONE questions for better reading and we have focused on the major and minor points of the reviewer*

1 - I think it would be better to use induction to explain how the expression of HERV-W is regulated by cytokines since you are saying that muscle repair is promoted by changes in the cytokine HERV-W, it would be better to explain what has been revealed about the relationship between cytokines and HERV-W.

*Authors’ Answer:* *We would like to thank you for the comment and, as suggested, we added a new paragraph in the "Discussion" section in order to improve the understanding of the relationship between cytokines and HERV-W, as presented below.*

*In the literature, it has been observed that the HERV-W family is not the only one capable of triggering the production of certain proinflammatory cytokines, such as IL-1β, IL-6, and TNF-α. This occurs through its interaction with Toll-like receptors (TLRs), particularly TLR4 and CD14. These interactions indicate that HERV transactivation can act as a factor fueling inflammation. Moreover, HERVs can activate the immune response, particularly innate immunity, leading to uncontrolled inflammation that may progress toward chronic inflammation*[17]*, or even some HERV Env proteins, particularly derived by HERV-H, HERV-K, and HERV-FRD (Syncytin-2), could be involved in immunosuppressive aspects* [18]*, by induction of the anti-inflammatory cytokine IL-10* [19,20]*.* *Additionally, it has been demonstrated that TNF-α is capable of increasing the RNA expression of HERV-H, HERV-K, and HERV-W through TNF-α receptor signaling*[21]*. Together, these findings highlight a close interplay between HERVs and inflammation, with HERVs exhibiting both proinflammatory and immunosuppressive roles. Lines (379-392).*

2- It would be helpful to provide the rationale for why the time point was set at 45 minutes. I think signals of muscle repair from exercise should be visible for several days, but it would be helpful to provide evidence that it is best to see them after 45 minutes with your chosen training method.

*Authors’ Answer*: *We would like to thank you for the comment and, first of all, we agree that muscle repair signals from exercise can be assessed for several hours/days. In addition, as suggested, we added a new paragraph in the "Discussion" section in order to clarify the reasons for assessing the study parameters after 45 minutes of the exercise session ends, as presented below.*

*It is worth mentioning that, in agreement with the literature, not only an acute exercise session is able to elicit several gene transcriptions but also it was reported that after 45 minutes more than 50% of these genes can be upregulated* [22]*. Specifically, resistance training can activate important signaling pathways 45 minutes after a bout of this exercise type as compared with rest. Moreover, it was reported that, at this time point, both restoration of tissue homeostasis and integrity, and also protein synthesis and degradation are still being regulated* [23] *and, interestingly, the human skeletal muscle is more sensitive to acute resistance training in the trained than untrained individuals, which reinforce the adaptive process that occurs in response to repeated stimulation* [24]*. These pieces of information may help us to understand the great changes that can be observed between 45 to 60 minutes after a bout of exercise in some molecules released in response to muscle contracting, such as IL-6 and IL-10* [25] *(Lines 362-373)*

3- I think it would be better to clearly state whether the exercises in this method are eccentric or concentric, if possible. This is because I think there is a distinction between eccentric and concentric movements when discussing their effects.

*Authors’ Answer:* *We thank the reviewer for the observation, we have added in the methods the characterization of the performed exercises in the methods section (Lines 162-164). We have also discussed this matter in the discussion session as presented below:*

*Corroborating this last suggestion, it was suggested that eccentric exercise does not necessarily elicit a larger increment of plasmatic IL-6 levels than concentric “nondamaging” muscle contractions, which not only shows that the elevation of systemic IL-6 is not closely associated with muscle damage but also eccentric exercise can lead to a delayed peak, as well as a slow dropped of this myokine in the recovery period* [26] *(Lines 418-423).*

*Specifically in the physical exercise context, it was demonstrated that TNF-α levels (both the mRNA in skeletal muscle and protein in the circulation) are, acutely, most prominently increased after the eccentric exercise than after concentric exercise* [27] *(Lines 429-432).*

4- Syncytin-1 is mentioned in the introduction and discussion, but if it is important for the hypothesis of MREV-W expression and muscle repair in this experiment, it would be better to show data on the expression of syncytin-1 as well. think. That will be more persuasive.

*Authors’ Answer: We thank the reviewer for this observation and we understand the reviewer concern. In fact, Syncytin-1 encoded by a particular HERV-W env gene, in the chromosome 7q21.2. It was showed that other loci of HERV-W envelope, homologue to Syncytin-1, are also overexpressed under inflammatory stress, but with distinct fusogenic capacities. But, despite of it, HERV-W envelope still have an immuno modulatory activity, due to the presence of a putative immunosuppressive domain (ISD)[28]. In a previous paper, also published in PlosOne, Sebastian Frese and colleagues [15] showed that not only the fusogenic envelope proteins as Syn-1, Syn-2 and 3 are expressed but also non-fusogenic HERV-envelope genes, suggesting additional roles for this highly immunogenic protein in muscular repair and inflammation.*

*Hence, focusing our discussion solely on Syncytin-1 activity may confuse our readers and potentially mislead the information provided. To address this concern, we have clearly stated in the discussion section the limitations of our study. Furthermore, we have removed most of the information regarding Syncytin-1 to prevent any potential misinformation.*

We hope that all our answers reach the reviewer and editor’s expectations. Please, do not hesitate if you have further questions.

Sincerely,

Luiz Henrique da Silva Nali

References

1.	Kim H-S, Kim D-S, Huh J-W, Ahn K, Yi J-M, Lee J-R, et al. Molecular characterization of the HERV-W env gene in humans and primates: expression, FISH, phylogeny, and evolution. Mol Cells. 2008;26: 53–60. Available: http://www.ncbi.nlm.nih.gov/pubmed/18525236

2.	Kim H-S, Lee W-H. Human Endogenous Retrovirus HERV-W Family: Chromosomal Localization, Identification, and Phylogeny. AIDS Res Hum Retroviruses. 2001;17: 643–648. doi:10.1089/088922201300119752

3.	Tristem M. Identification and characterization of novel human endogenous retrovirus families by phylogenetic screening of the human genome mapping project database. J Virol. 2000;74: 3715–30. Available: http://www.pubmedcentral.nih.gov/articlerender.fcgi?artid=111881&tool=pmcentrez&rendertype=abstract

4.	Voisset C, Blancher A, Perron H, Mandrand B, Mallet F, Paranhos-Baccala G. Phylogeny of a Novel Family of Human Endogenous Retrovirus Sequences, HERV-W, in Humans and Other Primates. AIDS Res Hum Retroviruses. 1999;15: 1529–1533. doi:10.1089/088922299309810

5.	Wang C, Zhai X, Wang S, Zhang B, Yang C, Song Y, et al. Comprehensive characterization of ERV-K (HML-8) in the chimpanzee genome revealed less genomic activity than humans. Front Cell Infect Microbiol. 2024;14. doi:10.3389/FCIMB.2024.1349046

6.	Flockerzi A, Burkhardt S, Schempp W, Meese E, Mayer J. Human endogenous retrovirus HERV-K14 families: status, variants, evolution, and mobilization of other cellular sequences. J Virol. 2005;79: 2941–2949. doi:10.1128/JVI.79.5.2941-2949.2005

7.	Romano CM, de Melo FL, Corsini MAB, Holmes EC, Zanotto PM d. A. Demographic Histories of ERV-K in Humans, Chimpanzees and Rhesus Monkeys. PLoS One. 2007;2. doi:10.1371/JOURNAL.PONE.0001026

8.	Lee A, Nolan A, Watson J, Tristem M. Identification of an ancient endogenous retrovirus, predating the divergence of the placental mammals. Philos Trans R Soc Lond B Biol Sci. 2013;368. doi:10.1098/RSTB.2012.0503

9.	Jha AR, Pillai SK, York VA, Sharp ER, Storm EC, Wachter DJ, et al. Cross-sectional dating of novel haplotypes of HERV-K 113 and HERV-K 115 indicate these proviruses originated in Africa before Homo sapiens. Mol Biol Evol. 2009;26: 2617–2626. doi:10.1093/MOLBEV/MSP180

10.	Quigley BL, Melzer A, Ellis W, Tzipori G, Nilsson K, Olagoke O, et al. Koala Retrovirus in Northern Australia Shows a Mixture of Stable Endogenization and Exogenous Lineage Diversification within Fragmented Koala Populations. J Virol. 2021;95. doi:10.1128/JVI.02084-20

11.	Ishida Y, Zhao K, Greenwood AD, Roca AL. Proliferation of endogenous retroviruses in the early stages of a host germ line invasion. Mol Biol Evol. 2015;32: 109–120. doi:10.1093/MOLBEV/MSU275

12.	Mi S, Lee X, Li X, Veldman GM, Finnerty H, Racie L, et al. Syncytin is a captive retroviral envelope protein involved in human placental morphogenesis. Nature. 2000;403: 785–9. doi:10.1038/35001608

13.	Ting CN, Rosenberg MP, Snow CM, Samuelson LC, Meisler MH. Endogenous retroviral sequences are required for tissue-specific expression of a human salivary amylase gene. Genes Dev. 1992;6: 1457–65. Available: http://www.ncbi.nlm.nih.gov/pubmed/1379564

14.	Medstrand P, Landry JR, Mager DL. Long terminal repeats are used as alternative promoters for the endothelin B receptor and apolipoprotein C-I genes in humans. J Biol Chem. 2001;276: 1896–903. doi:10.1074/jbc.M006557200

15.	Frese S, Ruebner M, Suhr F, Konou TM, Tappe KA, Toigo M, et al. Long-term endurance exercise in humans stimulates cell fusion of myoblasts along with fusogenic endogenous retroviral genes in vivo. PLoS One. 2015;10. doi:10.1371/journal.pone.0132099

16.	Søe K, Andersen TL, Hobolt-Pedersen AS, Bjerregaard Bolette B, Larsson LI, Delaissé JM. Involvement of human endogenous retroviral syncytin-1 in human osteoclast fusion. Bone. 2011;48: 837–846. doi:10.1016/J.BONE.2010.11.011

17.	Hurst TP, Magiorkinis G. Activation of the innate immune response by endogenous retroviruses. J Gen Virol. 2015;96: 1207–1218. doi:10.1099/JGV.0.000017

18.	Mangeney M, Renard M, Schlecht-Louf G, Bouallaga I, Heidmann O, Letzelter C, et al. Placental syncytins: Genetic disjunction between the fusogenic and immunosuppressive activity of retroviral envelope proteins. Proc Natl Acad Sci U S A. 2007;104: 20534–20539. doi:10.1073/PNAS.0707873105

19.	Denner J. The transmembrane proteins contribute to immunodeficiencies induced by HIV-1 and other retroviruses. AIDS. 2014;28: 1081–1090. doi:10.1097/QAD.0000000000000195

20.	Morozov VA, Dao Thi VL, Denner J. The transmembrane protein of the human endogenous retrovirus--K (HERV-K) modulates cytokine release and gene expression. PLoS One. 2013;8. doi:10.1371/JOURNAL.PONE.0070399

21.	Johnston JB, Silva C, Holden J, Warren KG, Clark AW, Power C. Monocyte activation and differentiation augment human endogenous retrovirus expression: implications for inflammatory brain diseases. Ann Neurol. 2001;50: 434–442. doi:10.1002/ANA.1131

22.	Hjorth M, Norheim F, Meen AJ, Pourteymour S, Lee S, Holen T, et al. The effect of acute and long-term physical activity on extracellular matrix and serglycin in human skeletal muscle. Physiol Rep. 2015;3. doi:10.14814/PHY2.12473

23.	Kumar V, Atherton P, Smith K, Rennie MJ. Human muscle protein synthesis and breakdown during and after exercise. J Appl Physiol. 2009;106: 2026–2039. doi:10.1152/JAPPLPHYSIOL.91481.2008

24.	Gehlert S, Weinisch P, Römisch-Margl W, Jaspers RT, Artati A, Adamski J, et al. Effects of Acute and Chronic Resistance Exercise on the Skeletal Muscle Metabolome. Metabolites. 2022;12. doi:10.3390/METABO12050445

25.	Ringleb M, Javelle F, Haunhorst S, Bloch W, Fennen L, Baumgart S, et al. Acute resistance exercise-induced changes in IL-6, IL-10, and IL-1ra in healthy adults: a systematic review and meta-analysis. medRxiv. 2023; 2023.05.10.23289790. doi:10.1101/2023.05.10.23289790

26.	Pedersen BK, Febbraio MA. Muscle as an endocrine organ: focus on muscle-derived interleukin-6. Physiol Rev. 2008;88: 1379–1406. doi:10.1152/PHYSREV.90100.2007

27.	HK V, S P, R C, D A, AN S, Z LA, et al. Acute Effects of Enhanced Eccentric and Concentric Resistance Exercise on Metabolism and Inflammation. J Nov Physiother. 2014;4. doi:10.4172/2165-7025.1000200

28.	Grandi N, Tramontano E. HERV Envelope Proteins: Physiological Role and Pathogenic Potential in Cancer and Autoimmunity. Front Microbiol. 2018;9. doi:10.3389/FMICB.2018.00462
